# Clinical utility of C-reactive protein to predict treatment response during cystic fibrosis pulmonary exacerbations

**DOI:** 10.1371/journal.pone.0171229

**Published:** 2017-02-08

**Authors:** Ashutosh Sharma, Gordon Kirkpatrick, Virginia Chen, Kate Skolnik, Zsuzsanna Hollander, Pearce Wilcox, Bradley S. Quon

**Affiliations:** 1 Department of Medicine, Centre for Heart Lung Innovation, University of British Columbia and St. Paul’s Hospital, Vancouver, British Columbia, Canada; 2 Department of Medicine, Division of Respiratory Medicine, University of British Columbia, Vancouver, British Columbia, Canada; 3 Prevention Of Organ Failure (PROOF) Centre of Excellence, Vancouver, British Columbia, Canada; 4 Department of Medicine, Division of Respiratory Medicine, University of Calgary, Calgary, Alberta, Canada; Children's Hospital of Los Angeles, UNITED STATES

## Abstract

**Rationale:**

C-reactive protein (CRP) is a systemic marker of inflammation that correlates with disease status in cystic fibrosis (CF). The clinical utility of CRP measurement to guide pulmonary exacerbation (PEx) treatment decisions remains uncertain.

**Objectives:**

To determine whether monitoring CRP during PEx treatment can be used to predict treatment response. We hypothesized that early changes in CRP can be used to predict treatment response.

**Methods:**

We reviewed all PEx events requiring hospitalization for intravenous (IV) antibiotics over 2 years at our institution. 83 PEx events met our eligibility criteria. CRP levels from admission to day 5 were evaluated to predict treatment non-response, using a modified version of a prior published composite definition. CRP was also evaluated to predict time until next exacerbation (TUNE).

**Measurements and main results:**

53% of 83 PEx events were classified as treatment non-response. Paradoxically, 24% of PEx events were characterized by a ≥ 50% increase in CRP levels within the first five days of treatment. Absolute change in CRP from admission to day 5 was not associated with treatment non-response (p = 0.58). Adjusted for FEV_1_% predicted, admission log_10_ CRP was associated with treatment non-response (OR: 2.39; 95% CI: 1.14 to 5.91; p = 0.03) and shorter TUNE (HR: 1.60; 95% CI: 1.13 to 2.27; p = 0.008). The area under the receiver operating characteristics (ROC) curve of admission CRP to predict treatment non-response was 0.72 (95% CI 0.61–0.83; p<0.001). 23% of PEx events were characterized by an admission CRP of > 75 mg/L with a specificity of 90% for treatment non-response.

**Conclusions:**

Admission CRP predicts treatment non-response and time until next exacerbation. A very elevated admission CRP (>75mg/L) is highly specific for treatment non-response and might be used to target high-risk patients for future interventional studies aimed at improving exacerbation outcomes.

## Introduction

Individuals with cystic fibrosis (CF) are prone to pulmonary exacerbations (PEx) with 45% of adults and 25% of children experiencing at least one event per year requiring IV antibiotics [1]. While there remains no gold standard definition of a PEx, these events are generally characterized by an increase in respiratory symptoms (e.g. cough, sputum production) beyond day-to-day fluctuation and a decline in lung function (i.e. FEV_1_) necessitating additional treatment [2]. Pulmonary exacerbations have significant implications, as they are associated with reduced quality of life [3], irreversible loss in lung function in up to 25% of cases [4, 5], and increased mortality [6, 7].

Regardless of the underlying trigger, uncontrolled infection and inflammation play important roles in the pathophysiology of PEx in CF [8]. Both airway and systemic inflammatory responses are exuberant upon clinical presentation with a PEx and decrease significantly in response to antibiotic therapy [9–12]. A number of blood-based biomarkers have been studied in the context of CF PEx and although none are exclusive to CF–related inflammation, a few have been found to be more strongly associated with PEx than other markers [9–12]. C-reactive protein (CRP) is a non-specific acute phase reactant that can be readily measured in most clinical laboratories and has been investigated the most extensively in CF [9]. Numerous studies have demonstrated that CRP is elevated at the onset of a PEx requiring IV antibiotics and decreases significantly by the end of treatment, corresponding to clinical improvement based on physician assessment [9, 10, 12–23].

While CRP levels decrease from beginning to end of treatment, no studies to date have characterized CRP levels early during the course of exacerbation treatment to determine if measures of systemic inflammation can be used to identify treatment non-responders sooner. The purpose of this study was to determine whether CRP monitoring early during PEx treatment could be used to predict treatment non-responders by the end of treatment. We hypothesized that the extent of CRP change from admission to day 5 of treatment would be predictive of treatment response. A useful biomarker in this setting could provide the opportunity to intervene earlier to potentially modify the treatment course and improve patient outcomes.

## Methods

### Study population

A retrospective cohort of consecutive adults with CF admitted to St. Paul’s Hospital (Vancouver, Canada) between April 1, 2013 and March 31, 2015 for the treatment of a PEx were screened for eligibility. PEx events were physician-diagnosed based on the modified Fuchs criteria [2] necessitating IV antibiotics. Subjects were included if CRP levels were measured within 24 hours of admission (day 0) and day 5 of treatment and FEV_1_% predicted was measured at baseline (i.e. best FEV_1_% predicted in the 6 months prior to admission) and within 48 hours of IV antibiotic completion. Transplant recipients and subjects with active inflammatory conditions (e.g. rheumatoid arthritis and allergic bronchopulmonary aspergillosis) requiring chronic immunosuppressive therapy were excluded. For patients with multiple PEx during the study, all events that met eligibility criteria were included. Patients discharged from hospital to home IV antibiotics were also included if they satisfied eligibility criteria. This study was considered minimal risk and the Providence Health Care Research Ethics Review Board (UBC-PHC REB H14-01050) approved the protocol and waived the need for patient consent.

### Design and procedures

Clinical and treatment data were extracted from the patients’ electronic health records and clinic charts. The data was accessed using patient identifiers but the extracted data was de-identified and stored anonymously (S1 Dataset). PEx were treated according to standard of care and included airway clearance therapies and IV antibiotics selected based on prior clinical response and sputum microbiology. While most IV antibiotic courses were planned for 14 days, the CF physician decided upon the completion date based on clinical, lung function, and radiographic response.

As part of a standardized CF admission order set, bedside spirometry and bloodwork (including CRP) were performed routinely during the hospitalization on all admitted patients. Spirometric measurements were obtained within 48 hours of admission and completion of IV antibiotics according to ATS criteria [24]. Blood was collected in plasma EDTA tubes within 24 hours of admission and twice weekly (every Monday and Thursday) for CRP measurements until IV antibiotic completion as part of routine care. CRP was quantified in the clinical laboratory using the Cardiophase high-sensitivity CRP assay analyzed on the ADVIA Chemistry system (Siemens Healthcare Diagnostics Inc., Malvern, PA). Elevated CRP and white blood cell (WBC) levels were defined based on clinical laboratory reference cut-off values of 3.1 mg/L and 11.0 x 10^9^ cells/L, respectively. As this was a retrospective study using routinely collected clinical data, the treating physician had full access to the results during treatment.

PEx treatment non-response was based on a modified version of a prior published definition [25]: 1) failure of FEV_1_% predicted to recover to 90% of baseline by the end of treatment; 2) prolongation of IV antibiotic therapy > 20 days; 3) change in antibiotic regime during hospitalization due to lack of clinical response (not due to allergic reactions) based on CF physician judgment; and/or 4) early recurrent PEx (<45 days) requiring oral or IV antibiotic therapy. As some studies have defined FEV_1_ recovery based on the best FEV_1_ measured in the 3 months following PEx treatment relative to the best FEV_1_ in the 6 months prior to exacerbation [4, 26], baseline recovery (criterion 1) of the composite definition defined above was modified according to this definition in a sensitivity analysis.

Overall change in CRP levels from admission to end of IV antibiotics and the relationship with relative change in FEV_1_% predicted was examined. CRP levels were examined on days 0 and 5 to determine if early changes in CRP could be used to predict treatment non-response. Changes from day 0 to day 5 were chosen *a priori* following discussion with local CF experts, as this would provide an opportunity to change treatment earlier than current practice. CRP levels on days 0 and 5 were also evaluated to predict treatment non-response. The above procedures were also repeated replacing CRP with WBC count as the predictive biomarker of interest to compare these two clinically available measurements.

We also determined whether CRP or WBC levels on day 0 or day 5 could be used to predict time until next exacerbation (TUNE). The TUNE event was determined based on the first PEx event requiring the use of oral or IV antibiotics following the end of a treated PEx event. The latest date of follow-up was December 31, 2015.

### Statistical analysis

Statistical analyses were performed using STATA 12.0 (StataCorp, Texas, USA) and R 3.2.3. Descriptive statistics including mean, standard deviation, range, and proportions were used as appropriate to summarize clinical and exacerbation characteristics. Spearman’s correlation was used to examine the association between variables, and p-values of less than 0.05 were considered to be statistically significant. CRP measurements were positively skewed and therefore the data was log_10_ transformed as the data was positively skewed but the WBC measurements were not transformed as the data was normally distributed. To compare admission and IV antibiotic completion measurements, a paired t-test was used.

Mixed effects logistic regression was performed (R package ‘lme4’) to assess the relationship between biomarker (CRP or WBC) and treatment response, accounting for the correlated nature of data for subjects with multiple PEx events. As patients with multiple PEx events could have biased our results, we performed several post-hoc sensitivity analyses. First, we restricted our analysis to patients with 1 or 2 PEx events. We also examined the correlation between admission CRP and TUNE in patients with frequent (≥ 2) vs. infrequent (< 2) PEx events. Lastly, we compared CRP levels at the various time points, baseline FEV_1_% predicted, and the proportion with non-response to treatment in patients with frequent vs. infrequent PEx events to determine if there were differences between the two groups. The following CRP and WBC parameters were evaluated: day 0, day 5, and the absolute change from day 0 to day 5. Cox proportional hazards models were used to determine if day 0 or day 5 CRP and WBC levels were associated with the risk of recurrent exacerbation requiring antibiotics (i.e. TUNE). All of the analyses were adjusted for baseline FEV_1_% predicted, as it was associated with CRP and treatment non-response and thus represents a confounder in our analysis.

To examine the prognostic performance of biomarkers significantly associated with treatment non-response based on logistic regression analysis, area under the receiver operating characteristics curve (ROC) curve (AUC) analysis was performed. The ROC curve was used to define cut-off values to optimize test sensitivity (≥ 90%) or specificity (≥ 90%).

## Results

### Baseline characteristics

**Fig 1:**
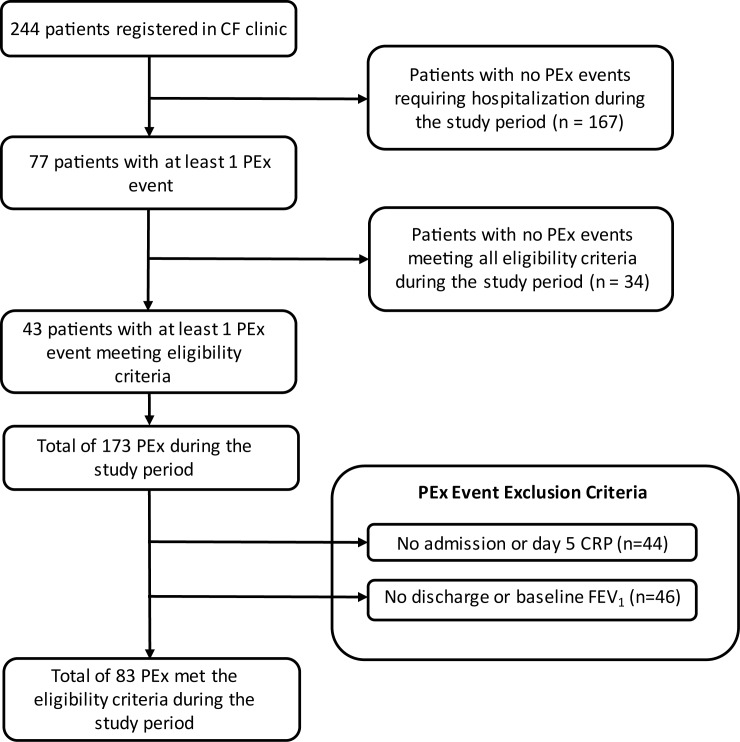
Flow diagram for cohort selection based on study inclusion and exclusion criteria.

Seventy-four unique patients were hospitalized for the treatment of a PEx during the study period. Forty-three patients met the eligibility criteria for at least one PEx and were thus included in the study (Fig 1). These 43 patients accounted for a total of 153 PEx events, of which 83 events met the study eligibility criteria. Included patients had a median of two PEx events meeting eligibility criteria during the study period. Most patients had moderate lung disease and the majority were chronically infected with *Pseudomonas aeruginosa* (Table 1).

**Table 1:** Clinical characteristics of included patients at the time of first PEx.

	N = 43
**Age, mean** ± **SD (range), yr**	33.7 ± 12.6 (19–70)
**Sex, female, n (%)**	22 (51%)
**Genotype, n (%)**	
Homozygous F508del	21 (49%)
Heterozygous F508del	14 (33%)
Other	8 (19%)
**Pancreatic insufficient, n (%)**	35 (81%)
**BMI, mean** ± **SD, kg/m**^**2**^	21.7 ± 3.5
**Baseline FEV**_**1**_**% predicted, mean** ± SD	58.9 ± 22.7
**FEV**_**1**_**% predicted group, n (%)**	
<40%	9 (22%)
40–70%	19 (44%)
>70%	15 (35%)
**Sputum microbiology, n (%)**	
*P*. *aeruginosa*	28 (65%)
MSSA	14 (33%)
MRSA	7 (16%)
*S*. *maltophilia*	2 (5%)
*B*. *cepacia complex*	2 (5%)
**CF-related diabetes, n (%)**	20 (47%)
**Chronic maintenance therapies, n (%)**	
Inhaled antibiotics	25 (58%)
Oral azithromycin	28 (65%)
Dornase alpha	28 (65%)
Hypertonics saline	16 (37%)
Inhaled corticosteroids	28 (65%)

Mean ± standard deviation or Number (proportion). **Abbreviations:** *B*. *cepacia complex* = *Burkholderia cepacia complex*, BMI = body mass index, FEV_1_ = forced expiratory volume in 1 second, IV = intravenous, kg/m^2^ = kilogram per metre-squared, MRSA = methicillin-resistant *Staphylococcus aureus*, MSSA = methicillin-sensitive *Staphylococcus aureus*, *P*. *aeruginosa* = *Pseudomonas aeruginosa*, *S*. *maltophilia* = *Stenotrophomonas maltophilia*

### PEx event characteristics

The mean duration of IV antibiotic therapy was 15.7 (SD 3.8) days (median 14; range, 8–33 d) and 95% of PEx events were treated with two or more IV antibiotics (Table 2). Five (6%) PEx events involved the use of systemic corticosteroids prior to (n = 2) or following (n = 3) admission for the treatment of overlapping asthmatic symptoms. From baseline to admission, mean FEV_1_% predicted decreased from 55.7 (23.5) % to 47.7 (22.4) %, with a mean difference of 8.0% (95% CI: 5.8–10.2; p = 0.0001). For 36% of PEx events, FEV_1_% predicted decreased by an absolute of ≥ 10% compared to baseline. From admission to antibiotic completion, FEV_1_% predicted increased by a mean of 6.5% (95% CI: 5.2–7.8; p = 0.0001).

**Table 2:** PEx treatment characteristics.

N = 83 exacerbation events	N	Admission	Day 5	End of Therapy	Mean Difference Admission to End of Therapy (95% CI)	P-value
**Plasma CRP (log**_**10**_ **transformed values), mean (SD), mg/L**	83	1.25 (0.69)	0.88 (0.56)	0.56 (0.51)	-0.69 (-0.55 to -0.82)	<0.0001
**Serum WBC, mean (SD), x 10**^**9**^ **cells/L**	83	11.1 (3.5)	8.2 (2.7)	8.3 (2.8)	-2.8 (-2.1 to -3.5)	<0.0001
**FEV**_**1**_**% predicted, mean (SD)**	66*	47.7 (22.4)	---	54.2 (24.2)	6.5 (5.2 to 7.8)	<0.0001
**IV antibiotic therapy used, n (%)**	83					
Ceftazadime		42 (51%)				
Meropenem		36 (43%)				
Tobramycin		34 (41%)				
Piperacillin-Tazobactam		16 (19%)				
Colistin		15 (18%)				
Aztreonam		12 (14%)				
Vancomycin		9 (11%)				
Cloxacillin		1 (1%)				

Mean (SD) or Number (proportion). **Abbreviations:** CRP = C-reactive protein, FEV_1_ = forced expiratory volume in 1 second, IV = intravenous, WBC = white blood cell

*17 events were missing an admission FEV_1_ measurement since this was not part of the study eligibility criteria

**Fig 2:**
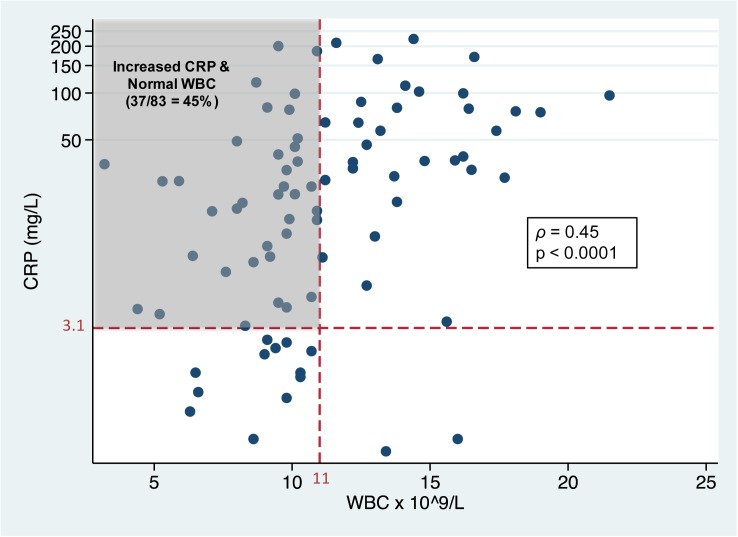
Scatterplot of admission (day 0) C-reactive protein (CRP) levels and WBC count. CRP and WBC are significantly correlated but 45% of pulmonary exacerbation events are characterized by an elevated CRP (>3.1 mg/L) and normal WBC (<11 x 10^9^ cells/L).

At admission, CRP levels were correlated with WBC levels (*ρ* = 0.45; p < 0.0001); however, 45% of PEx events were characterized by an elevated CRP level but a normal WBC count (Fig 2). On the contrary, an elevated WBC count and normal CRP characterized just two PEx events. PEx events with an elevated CRP level and normal WBC count had an absolute improvement in FEV_1_% predicted of 6.8 (5.4) % from beginning to end of IV antibiotic, which was similar to the improvement of 5.6 (4.8) % for PEx events with elevation of both admission CRP and WBC (p = 0.40).

PEx events in patients with lower baseline FEV_1_ values had higher admission CRP levels (*ρ* = -0.65; p < 0.0001) and WBC (*ρ* = -0.39; p = 0.0002) counts. There were no other relationships between admission CRP levels or WBC counts and other baseline characteristics (age, sex, or sputum microbiology).

### Longitudinal changes in CRP and WBC measurements

From admission to IV antibiotic completion, CRP levels decreased by a mean of 0.69 log_10_ (95% CI: 0.55 to 0.82; p < 0.0001) and WBC count decreased by a mean of 2.8 (95% CI: 2.1–3.5; p < 0.0001) (Table 2). Neither the magnitude of change in CRP or WBC from admission to IV antibiotic completion was correlated with the relative change in FEV_1_% predicted (both p > 0.05). Unexpectedly, 34 (41%) patients experienced an increase in CRP levels within the first 5 days of antibiotic treatment relative to admission, with 20 (24%) experiencing a CRP increase of at least 50%. In a *post-hoc* analysis, PEx factors independently associated with a CRP increase of ≥ 50% within the first 5 days of treatment included the use of IV colistin (OR 61.7; 95% CI 3.3–1138.2; p = 0.005) and IV piperacillin-tazobactam (OR 17.6; 95% CI 1.9–161.0; p = 0.01).

### CRP and WBC to predict PEx treatment non-response

A total of 44 (53%) PEx events were defined as treatment non-response; 21 (25%) of which were due to a failure of discharge FEV_1_ to reach 90% of baseline FEV_1_, 13 (16%) due to prolongation of antibiotic therapy, 5 (6%) due to a change in antibiotics regime, and 24 (29%) due to early PEx recurrence (< 45 days) requiring oral or IV antibiotics. One failure criterion was met for 28 PEx events, two for 13 PEx events, and at least three for 3 PEx events.

Baseline and admission FEV_1_% predicted were associated with treatment non-response (both p<0.05) in bivariate analysis but the associations were no longer significant in a multivariate model that included admission log_10_ CRP. Change in log_10_ CRP from admission to day 5 of treatment was not significantly associated with treatment non-response after adjustment for baseline FEV_1_% predicted (p = 0.58) (Table 3). Increase in CRP by ≥ 50% within the first 5 days was also not predictive of treatment non-response (p = 0.20). Log_10_ CRP levels on admission (day 0) were associated with treatment non-response after adjustment for baseline FEV_1_% predicted (aOR: 2.39; 95% CI: 1.14 to 5.91; p = 0.03). However, due to the smaller sample size, this association was no longer statistically significant when we excluded patients with frequent PEx events (> 2 PEx during the study period) in a post-hoc sensitivity analysis, however the effect size remained similar to our primary analysis (aOR: 1.90; 95% CI: 0.8 to 4.3; p = 0.14). There were no significant differences in CRP levels, baseline FEV_1_% predicted, or proportion of patients with treatment non-response in patients with frequent (≥ 2) vs. infrequent (< 2) PEx. Furthermore, the correlation between admission CRP and TUNE was significant for both infrequent (*ρ* = -0.60; p = 0.006) and frequent (*ρ* = -0.43; p = 0.0007) PEx groups.

**Table 3:** Evaluation of CRP and WBC to predict PEx treatment non-response.

		aOR* [95% CI]	P-value
**Log**_**10**_ **CRP**			
	**Admission (Day 0)**	2.39 (1.14 to 5.91)	0.03
	**Day 5**	2.02 (1.01 to 4.62)	0.06
	**Absolute change Day 0 to Day 5**	0.85 (0.45 to 1.55)	0.58
**WBC**			
	**Admission (Day 0)**	1.19 (0.65 to 2.34)	0.59
	**Day 5**	1.16 (0.66 to 2.01)	0.59
	**Absolute change Day 0 to Day 5**	0.94 (0.54 to 1.64)	0.83

*Adjusted for baseline FEV_1_% predicted

**Abbreviations:** aOR = adjusted odds ratio, CRP = C-reactive protein, FEV1 = forced expiratory volume in 1 second, WBC = white blood cell count

**Fig 3:**
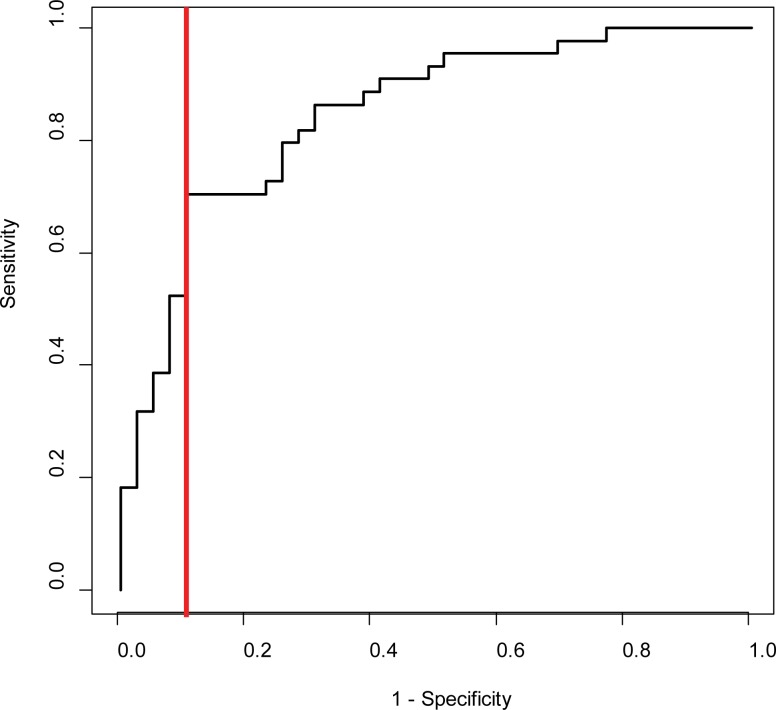
ROC curve for the prediction of treatment non-response based on admission log_10_ CRP adjusted for baseline FEV_1_% predicted. As can be seen from the ROC curve, a CRP cut-off of >75 mg/L corresponds to a test specificity of 90% and sensitivity of 70%.

The AUC of admission CRP to predict treatment non-response was 0.72 (95% CI 0.61–0.83; p<0.001). Approximately one-quarter (23%) of PEx events in our cohort were characterized by an admission CRP > 75 mg/L. Based on the ROC curve (Fig 3), an admission CRP of > 75 mg/L resulted in a specificity of 90% and sensitivity of 70% for the prediction of treatment non-response. Absolute change in WBC counts from day 0 to day 5, and WBC counts on day 0 and day 5, were not associated with treatment non-response (p > 0.05). In a sensitivity analysis, the 90% recovery of baseline lung function treatment response criterion was extended to include the best FEV_1_ at treatment completion or within 3 months of IV antibiotic completion as defined in prior studies [4, 26] but this did not alter the findings (data not shown).

### CRP and WBC to predict time until next exacerbation

The mean TUNE was 101 days (range: 9–311 days). Log_10_ CRP levels on day 0 (HR 1.60; 95% CI 1.13–2.27; p = 0.008) and day 5 (HR 1.63; 95% CI 1.17–2.27; p = 0.004) were significantly associated with TUNE, after adjusting for baseline FEV_1_% predicted. WBC counts on day 0 and day 5 were not associated with TUNE (p>0.05).

## Discussion

Exacerbation outcomes remain suboptimal with 25% of patients failing to recover their baseline lung function despite treatment [4, 27]. Therefore, biomarkers predictive of treatment non-response are urgently needed to guide more timely exacerbation treatment decisions and the selection of patients for interventional trials aimed at improving exacerbation outcomes [28]. Our results are consistent with previous studies demonstrating that CRP decreases from beginning to end of IV antibiotic treatment [9, 10, 12–22]. However, in contrast to our hypothesis, we did not find a significant association between early changes in CRP or WBC and treatment response.

There are a few potential explanations as to why early changes in CRP were not predictive of treatment response. First, we found that admission CRP levels inversely correlate with baseline FEV_1_% predicted. Therefore, the magnitude of absolute change in CRP from admission to day 5 depends not only upon changes in disease activity in response to treatment but also upon underlying disease severity. Second, the pattern of CRP change within the first 5 days of treatment was heterogeneous with 40% of PEx events characterized by an initial increase in CRP, and an increase exceeding 50% for nearly 25% of PEx events. This initial increase could have offset any expected decrease by day 5 thus diminishing its predictive value.

To our knowledge, this is the first study to report an increase in CRP levels early during PEx treatment; however, this early increase did not predict treatment non-response. CRP is a non-specific marker of systemic inflammation and can change with disease activity and from the effects of antibiotics. We postulated that CRP could have increased in a subset of patients due to the pro-inflammatory effects of bacterial endotoxins (e.g. LPS [29, 30]) and/or cell wall components (e.g. lipoteichoic acid [31]) released following bacterial killing. However, we did not observe an independent association between CRP increase and the type of infecting organism (*i*.*e*., gram positive *vs*. gram negative bacteria) to suggest this response was driven by a particular bacterial component. Alternatively, it could have increased due to a toxic effect or hypersensitivity reaction to the antibiotic itself. Interestingly, CRP was independently associated with the use of two specific IV antibiotics—colistin and piperacillin-tazobactam. Colistin can cause neurotoxicity and nephrotoxicity [32] and while no adverse events to this antibiotic were documented in this study, the toxic effects could have been subclinical. Nebulized formulations of colistin can elicit a robust airway inflammatory response [33] and by extension IV formulations could also have a similar effect on systemic inflammation.

While monitoring early changes in CRP during the PEx treatment course did not appear to be useful in predicting non-response, CRP measurements at admission or later in the PEx course could be useful. We found that a higher admission CRP was associated with treatment non-response and shorter TUNE. This finding is consistent with the results of a study by Parkins *et al*. [25]. Based on our results, an admission CRP of greater than 75 mg/L, which was present in about one-quarter of PEx events, demonstrated high specificity (90%) for the prediction of treatment non-response and might be used in future interventional CF PEx trials to target patients at high risk of treatment failure and who might benefit from short courses of systemic corticosteroids [34]. For example, CRP has been used in community-acquired pneumonia trials to identify patients with a higher systemic inflammatory response at admission, representing a subgroup potentially more likely to benefit from corticosteroids. In this context, patients randomized to methylprednisolone were less likely to experience treatment failure compared to placebo [35]. Interestingly, the absolute CRP values at the time of admission reported in our study involving CF patients are lower than the values reported in the aforementioned community-acquired pneumonia study, whereby the upper quartile of CRP values was > 150 mg/L [35]. This would be in keeping with the clinical presentation of CF PEx as infection is largely confined to the lung, with systemic involvement (e.g. bacteremia, sepsis) being uncommon relative to the presentation of community-acquired pneumonia in non-CF hosts. While not specifically examined in our study, at least two prior studies have also reported that a higher CRP later in the PEx course is associated with an increased risk of subsequent exacerbation [36, 37]. Future studies will need to determine whether altering or extending treatment if the CRP level remains elevated near the end of the PEx treatment course improves outcomes.

CRP is a more sensitive indicator of PEx “onset” compared to WBC count as 45% of PEx events were characterized by a normal WBC count but elevated CRP at the time of hospital admission. Physicians often rely upon an elevated WBC count as an indicator of acute infection in decisions to start antibiotics or not. Over-reliance on WBC count could lead to the underutilization of antibiotics in CF, especially as CF patients often appear “well” (especially to non-CF care providers) and radiographic changes are often subtle or absent during PEx. In this study, PEx events with normal WBC but elevated CRP experienced similar improvements in FEV_1_% predicted following IV antibiotics compared to events characterized by elevated WBC and CRP. This finding underscores the importance of measuring CRP if a PEx is suspected to reduce the risk of under-treatment.

As this was a retrospective study and CRP was measured as part of routine clinical assessment with the results un-blinded to the clinical team, CRP levels could have influenced clinician decision-making, thus inducing a relationship between CRP and treatment response. We do not believe this potential bias had a major impact on our results, as we did not identify an association between early change in CRP levels and treatment response. While we did identify an association between admission CRP and treatment non-response, it is unlikely that admission CRP would have influenced treatment decisions that were part of our composite outcome definition (such as duration of treatment or need for antibiotic switches) as they were decided upon much later in the PEx treatment course, but we cannot exclude this potential influence and bias.

There are a number of other potential limitations to this study. We used an adapted version of a previously published composite definition of treatment non-response [25] due to the lack of a gold standard for treatment response. This composite score did not include symptom response, which would have been important to evaluate but could not be collected due to the study’s retrospective design. Our treatment non-response rate was nearly double that reported by Parkins *et al*. as we modified their outcome definition to include the recurrent need for oral or IV antibiotics within 45 days (as opposed to IV antibiotics only), as we had access to oral antibiotic prescribing data and felt that capturing this was a more reliable indicator of non-response to therapy. Similar to other studies [4, 5, 38], 25% of patients in our study did not recover to within 90% of baseline lung function by the end of treatment and therefore we do not believe our higher non-response rates were due to the selection of sicker patients.

We had a relatively small number of PEx events, which limited our ability to examine and validate clinical factors associated with treatment non-response observed in prior studies [4, 25]. While the small sample size could have led to an underpowered analysis, we feel the risk of committing a type II error in not finding an association between change in CRP and treatment non-response is low based on the p-value not being close to achieving statistical significance. The inclusion of patients with frequent PEx (three or more) could have biased the association between admission CRP and non-response but based on the results of the post-hoc sensitivity analysis, the effect size was not substantially different when we restricted the analysis to patients with one or two exacerbations. Furthermore, there were no significant differences in CRP levels, baseline FEV_1_% predicted, or proportion of patients with treatment non-response for frequent vs. infrequent PEx groups.

There were low rates of complete data (58% of patients and 54% of PEx events) as we only included PEx events with admission and day 5 CRP measurements as well as baseline and discharge FEV_1_ measurements. While CRP measurements (2x per week) and discharge FEV_1_ are part of the standardized admission orders and therefore should have been available on all admitted patients, CRP measurements did not fall within 24 hours of admission and day 5 in some cases. Furthermore, not all academic house staff used the pre-set admission orders, especially early in the study period, due to lack of awareness as the admission orders were newly implemented. While lack of complete data may not be random in a clinical setting we did not find any significant difference (p = 0.62) in the baseline FEV_1_% predicted of patients who were included (mean 58.9; standard deviation 22.7) *vs*. excluded (mean 61.6; standard deviation 23.7) and therefore we do not believe selection bias is a major factor impacting the generalizability of our results. Including subjects requiring hospitalization could limit the generalizability of our findings but the majority of exacerbations requiring IV antibiotics at our centre are treated, at least initially, in hospital. Lastly, given the limited number of PEx events treated with prednisone (6%), we couldn’t examine the effects of prednisone on changes in CRP but this could be the focus of future studies examining the effects of prednisone on systemic inflammation and ultimate treatment response.

## Conclusions

CRP decreases significantly from beginning to end of exacerbation treatment but early changes in CRP are not predictive of treatment response. Admission CRP predicts treatment non-response and time until next exacerbation requiring oral or IV antibiotics and could be used to select patients for future interventional studies aimed at improving exacerbation outcomes.

## Supporting information

S1 Dataset De-identified clinical and CRP data used for data analysis. (XLSX)

## Abbreviations

ATS: American Thoracic Society
AUC: area under the curve
CI: confidence interval
CRP: C-reactive protein
EDTA: ethylenediaminetetraacetic acid
FEV_1_: forced expiratory volume in 1 second
HR: hazard ratio
IV: intravenous
LPS: lipopolysaccharide
OR: odds ratio
PEx: pulmonary exacerbations
ROC: receiver operating characteristics
SD: standard deviation
TUNE: time until next exacerbation
WBC: white blood cell
